# PEN-DEL: implementing penicillin allergy de-labeling in hospitalized older adults – a quality improvement initiative

**DOI:** 10.1017/ash.2025.10279

**Published:** 2026-02-10

**Authors:** Danielle Grace Co, Merisa Mok, Shirin Malek, Gary Kwan, Vincent H. Mabasa, Laurenna Peters, Kevin Afra

**Affiliations:** 1 Lower Mainland Pharmacy Services, Burnaby Hospital, Fraser Health Authority, Burnaby, BC, Canada; 2 Faculty of Pharmaceutical Sciences, University of British Columbia, Vancouver, BC, Canada; 3 Division of Infectious Diseases, Department of Medicine, Fraser Health Authority, Burnaby, BC, Canada; 4 Division of Infectious Diseases, Department of Medicine, University of British Columbia, Vancouver, BC, Canada

## Abstract

**Background:**

Erroneously labeled penicillin allergies can lead to prolonged hospitalization, increased adverse effects and infection rates with alternative antibiotics. Although elderly patients are more vulnerable to these negative outcomes, penicillin allergy assessments and de-labeling remain underutilized in this population.

**Objective:**

To assess the efficacy and challenges of implementing penicillin allergy de-labeling in hospitalized elderly patients.

**Methods:**

Between March 2024 and April 2025, we conducted a quality improvement study on patients who were 65 years and older, had a penicillin allergy, and were admitted to Burnaby Hospital Acute Care for Elderly (ACE) Unit. Patients were proactively screened, interviewed, and assessed for eligibility in allergy de-labeling based on PEN-FAST score ≤1, predefined criteria, clinical judgment, and patient consent. If penicillin challenge was given, patients received pharmacist follow-up and 4-week outcomes were documented. A post-implementation survey was distributed to ward healthcare staff to identify barriers to implementation.

**Results:**

Out of 105 patients screened, 87 patients met study inclusion criteria. Among 34 patients deemed eligible for de-labeling, 24 patients (71%) were de-labeled from either oral amoxicillin challenge or information gathering alone. Within four weeks after de-labeling, 10 patients (42%) received beta-lactam antibiotics, and no patients received guideline-discordant broad-spectrum antibiotics. Only 1 patient experienced a delayed reaction to amoxicillin-clavulanate. From surveying ACE unit nursing, physician, and pharmacy staff, frequently reported barriers to allergy assessment and de-labeling included time and staffing constraints, and patient complexity.

**Conclusions:**

Our pharmacy-driven interdisciplinary penicillin allergy de-labeling initiative is effective and safe for eligible hospitalized older adults with low PEN-FAST scores (≤1).

## Introduction

Although penicillin allergy is frequently reported in about 10% in the general population, 90% of these reported allergies are not true allergies based on penicillin skin testing.^1–5^ Many of these allergies are misinterpreted as they typically originate from childhood, persist and remain unverified, and may be the result of side effects or infectious symptoms as opposed to true allergic reactions.^6^ Moreover, true IgE-mediated penicillin allergies can often diminish over time.^7^ Mislabeled penicillin allergies contribute to longer hospital stays, increased adverse effects and increased infection rates with alternative antibiotics (eg, methicillin-resistant *Staphylococcus aureus*, *Clostridioides difficile*), and greater healthcare costs.^8–13^ These outcomes are particularly concerning for the elderly, whom up to 16% have penicillin allergies and are more vulnerable to infections and adverse drug reactions.^14–17^

To mitigate the negative impact of erroneous penicillin allergy labels, initiatives are needed to assess and de-label these allergies. The PEN-FAST tool, a validated scoring system, can stratify adult patients by penicillin allergy risk (ie, the risk of having a positive penicillin allergy test).^18^ Patients deemed “low-risk” may safely undergo a direct oral penicillin challenge without resource-intensive skin testing.^19^ While this approach has been implemented in various settings such as emergency departments, allergy clinics, preoperative clinics, intensive care units, and Veteran Affairs populations, this approach remains underutilized in hospitalized older adults.^14,20–27^

In Spring 2024, Burnaby Hospital launched a pharmacy-driven interdisciplinary penicillin allergy de-labeling quality improvement initiative in the Acute Care for Elderly (ACE) Unit. The ACE unit primarily provides care to older adults with multiple medical conditions.^28^ Prior to this initiative, there was no formal penicillin allergy assessment program in place. We conducted a quality improvement study to assess the feasibility, safety, and challenges of implementing this initiative in the ACE Unit.

## Methods

### Allergy screening and de-labeling initiative

This pilot initiative was conducted at a large community university-affiliated hospital with around 300 beds in Burnaby, British Columbia, Canada.^29,30^ The hospital has infectious diseases physician and antimicrobial stewardship (AMS) teams working on site. The hospital does not have access to on-site allergy and immunology staff.

The penicillin allergy assessment process begins with the clinical pharmacy support technician (CPST) using electronic medical record (EMR) on weekdays to identify patients with a documented penicillin allergy. The CPST collaborated with ward and AMS clinical pharmacists to interview patients and gather detailed allergy histories using a locally developed standardized work-up form (Supplementary Material 1). Clinical pharmacists reviewed these patients. Piperacillin-tazobactam allergy patients were excluded from the de-labelling initiative.

If information gathering alone was sufficient for de-labeling (ie, reaction was intolerance, or low-risk patient had PEN-FAST score of 0 or 1 with subsequent penicillin tolerance history (not based on piperacillin-tazobactam tolerance alone)), the patient was de-labeled, educated, and allergy records were updated accordingly.

For patients with a PEN-FAST score of 0 or 1 and no contraindications to oral penicillin challenge (Supplementary Material 1), the patient’s medical and pharmacy team offered a single 500 mg dose of oral amoxicillin. Oral penicillin challenge was not pursued for patients with poor prognosis or if it did not align with their goals of care. Patients with higher-risk histories who did not qualify for de-labeling (eg, multiple or recurrent allergic reactions) were referred to an allergist for outpatient follow-up. Prior to each penicillin challenge procedure, patient consent was obtained and the health authority protocol was used. If patients had received antihistamines in the last 5 days, or beta-blockers on the morning of challenge, oral penicillin challenge was rescheduled. Vital signs were monitored by nursing staff pre-challenge and every 30 minutes for at least 1 hour after oral challenge. If no reaction occurred, patients were de-labeled, counseled, and their allergy records were updated as having tolerated the penicillin challenge. After 4 weeks, the pharmacist followed up with the patient to check for any delayed reactions and to inquire about any antimicrobial use. Any new reactions deemed to be an allergic reaction led to reclassification of the allergy records.

### Study design, approval and eligibility criteria

This quality improvement study involved a retrospective chart review of patients assessed during the penicillin allergy de-labeling pilot initiative, and a post-implementation staff survey. The quality improvement study was granted research ethics board review exemption and survey review from the health authority research ethics board. Patients aged 65 years and older and admitted to the ACE Unit between March 25, 2024 and April 17, 2025 were included in the study. Patients were excluded if they had been discharged prior to full allergy assessment or had piperacillin-tazobactam allergy.

### Survey

A post-implementation survey, adapted from the Theoretical Domains Framework, was distributed by the ward pharmacist to ACE Unit staff including pharmacists, pharmacy technicians, nurses, and physicians from December 2024 to January 2025.^31–35^ Paper and electronic forms were made available for staff to complete anonymously. Survey items included themes consistent with previously identified barriers to practice change in healthcare such as knowledge, skills, social and professional role and identity, beliefs about capabilities, beliefs about consequences, motivation, and environmental context and resources. Demographic questions, Likert scale, multiple choice, and open-ended question formats were utilized (Supplementary Material 2).

### Outcomes

Our primary outcome was the proportion of eligible patients whose penicillin allergies were de-labeled. We also investigated the following secondary outcomes: method of de-labeling (ie, information gathering alone or oral penicillin challenge), immediate and delayed reactions, beta-lactam use within 4 weeks, guideline-discordant broad-spectrum antibiotic use within 4 weeks, allergy record updates, and healthcare staff reported barriers to this initiative.

### Data collection

To collect data, we used the EMR and pharmacy penicillin allergy work-up form (Supplementary Material 1). Characteristics include age, sex, comorbidities, length of hospital stay, whether they had an infection during admission, and pertinent details of their index penicillin reaction or drug allergy history. Penicillin allergy history details include the type of penicillin antibiotic the patient experienced a reaction to, how long ago the reaction occurred, signs and symptoms experienced, time to reaction following administration, whether the penicillin was stopped, whether treatment was required, whether the patient tried the same or another penicillin antibiotic after the reaction, and whether they had previous penicillin skin testing done. PEN-FAST scores were also calculated. Baseline characteristics of survey respondents including profession, years of practice, and reported barriers to implementation were collected (Supplementary Material 2).

### Statistical analysis

Descriptive statistics were calculated using Microsoft Excel (Version 2402). Frequencies were reported for categorical variables and medians with interquartile ranges (IQRs) were calculated for continuous variables.

## Results

**Figure 1.**
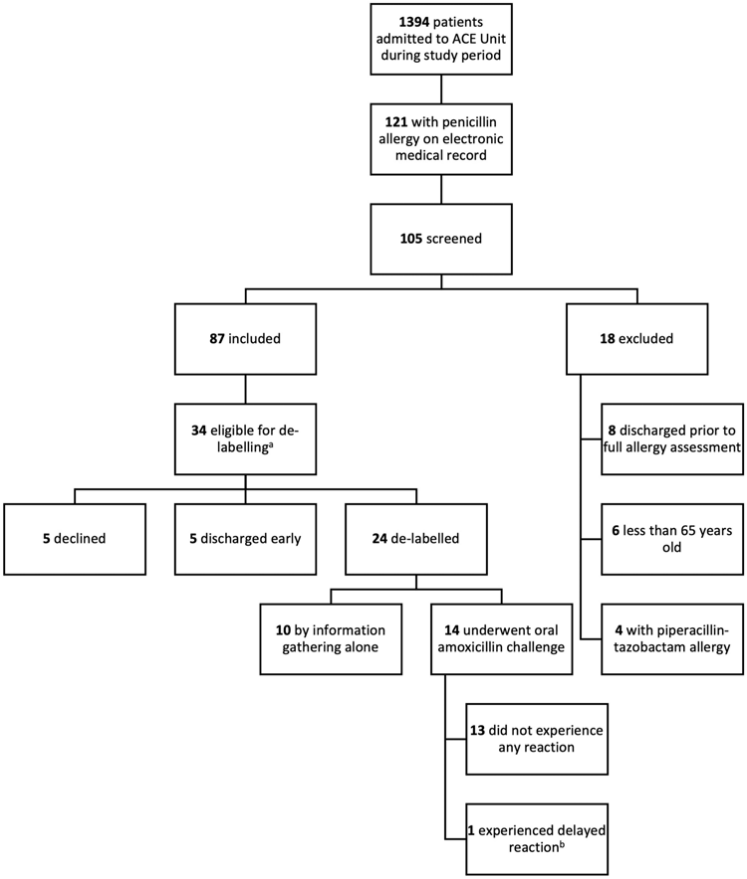
Flow diagram of patients included in the study. ^a^According to PEN-FAST score of 0 or 1, predefined criteria, and providers’ clinical judgment. ^b^Patient tolerated oral amoxicillin challenge and was subsequently transitioned to oral amoxicillin-clavulanate but experienced a delayed reaction (rib cage tightness, pain upon breathing, coughing, itchy rash on back) after 7 days of treatment. Rib cage tightness/pain resolved after 2 doses of antihistamine and itchy rash on back resolved after 1 week of antihistamines.

From March 25, 2024 to April 17, 2025, a total of 1,394 patients were admitted to the ACE Unit. Of these, 121 patients (8.7%) had a penicillin allergy. Out of 105 patients screened during weekday working hours, a total of 87 patients met study inclusion. Reasons for exclusion included early discharge, age less than 65 years, and piperacillin-tazobactam allergy (Figure 1).

### Characteristics of included patients

Baseline patient demographics are outlined in Table 1. The median age was 84 years (IQR 78–90) with the majority being female (75%). The median Charlson Comorbidity Index was 6 (IQR 5–8). Unspecified penicillin allergies (72%) accounted for most reported penicillin allergies, followed by penicillin VK (15%) and amoxicillin (9%). The most frequently reported reaction to penicillin was rash (37%). Most reactions were reported to have occurred greater than 10 years ago (67%). The median PEN-FAST score for these patients was 1 (IQR 0–2), with 34% of study patients having a PEN-FAST score of 2 or more.

**Table 1. tbl1:** Patient baseline characteristics

Characteristic	*n* = 87
Age, years, median (IQR)	84 (78–90)
Age groups, *n* (%)	
65–74	11 (13)
75–84	34 (39)
85–94	32 (37)
≥95	10 (11)
Female, *n* (%)	65 (75)
Charlson Comorbidity Index, median (IQR)	6 (5–8)
Length of hospital stay, median (IQR), days	16 (9–29)
Reported index penicillin allergy label, *n* (%)^a^	
Penicillin unspecified	63 (72)
Penicillin VK	13 (15)
Penicillin G	5 (6)
Amoxicillin	8 (9)
Ampicillin	3 (3)
Amoxicillin-clavulanate	2 (2)
Cloxacillin	1 (1)
Timeline of index penicillin reaction, *n* (%)	
≤5 years ago	3 (3)
6–10 years ago	5 (6)
>10 years ago	58 (67)
Unknown	15 (17)
Patient denied penicillin allergy	6 (7)
Time to reaction following index penicillin administration, *n* (%)	
Within hours	27 (31)
By 3–7 days	2 (2)
>7 days	3 (3)
Unknown	49 (56)
Patient denied penicillin allergy	6 (7)
Reported penicillin reaction, *n* (%)^a^	
Skin rash	32 (37)
Gastrointestinal	1 (1)
Anaphylaxis	20 (23)
Delayed severe reaction	5 (6)
• Type II	2 (2)
• Type III	1 (1)
• Type IV	2 (2)
Other^b^	5 (6)
Unknown	31 (36)
Patient denied penicillin allergy	6 (7)
Treatment required for penicillin reaction, *n* (%)	
Yes	6 (7)
• Antihistamine	0
• Epinephrine	3 (3)
• Corticosteroid	1 (1)
• Hospitalization	2 (2)
No	20 (23)
Unknown	55 (63)
Positive penicillin skin test, *n* (%)	
Yes	2 (2)
No	1 (1)
Not done	67 (77)
Unknown	17 (20)
PEN-FAST score, *n* (%)	
0	24 (28)
1	33 (38)
2	10 (11)
3	19 (22)
4	0
5	1 (1)
Median PEN-FAST score (IQR)	1 (0–2)
For PEN-FAST 0–1, contraindications to oral amoxicillin challenge, *n*	
NPO or unable to take oral medications	1
Use of immunosuppressives	3
Significant cardiovascular disease (including active ACS in last 6 months)	1
Current oxygen supply ≥2L/min (not at baseline)	0
Hemodynamically unstable	1
Active, uncontrolled asthma or COPD	1
Chronic spontaneous urticaria/mast cell disease	0
Other (recurrent or Type II-IV reactions, allergy to multiple beta-lactams, acute delirium, concurrent rash)	7
None	41
Patients referred to allergist, *n*	6

See (Supplementary Material 3) for additional baseline characteristics

NPO - nothing by mouth; ACS—acute coronary syndrome; COPD—chronic obstructive pulmonary disease.

a Percentages may add up to greater than 100% as patients may have had more than one allergy or reaction listed.

b Reported reactions: “fainting”, “tingling, cold feeling”, and “high fever up to 42°C with hallucinations”.

### Penicillin allergy de-labeling

Thirty-four of the 87 included study patients (39%) were deemed eligible for penicillin allergy de-labeling based on PEN-FAST score, clinical judgment, and without contraindications to oral challenge (Figure 1). Of the eligible patients, five patients declined consent and five patients were discharged prior to obtaining consent. The remaining patients were de-labeled (24 of 34, 71%) (Table 2).

Of the 24 eligible patients who were de-labeled, the de-labeling method was based on history (*n* = 10; 42%) or oral amoxicillin challenge (*n* = 14; 58%). No immediate reactions were reported post-challenge. One patient reported developing a delayed reaction after 7 days. The patient had rib cage tightness, pain upon breathing, and coughing without shortness of breath which led to discontinuation of the antibiotic. On the eighth day, the patient developed an extensive itchy rash on their back. The rib cage tightness and pain resolved after 2 doses of diphenhydramine and the itchy rash on the back resolved after 1 week of diphenhydramine. The patient’s family physician and community pharmacy were notified, and the patient’s allergy records were reverted to having a penicillin allergy with reaction details.

Among the patients who received de-labeling, 42% of patients received beta-lactam antibiotics within 4 weeks. No patients received guideline-discordant broad-spectrum antibiotics within 4 weeks.

### Allergy record updates

Eighty-five of 87 patients (98%) had their local health authority allergy records updated during this initiative, while 2 patients did not have any new information to add to their existing record. When applicable, requests were submitted to update provincial, outpatient pharmacy, and family physician clinic allergy records.

### Survey to ACE unit staff

**Figure 2.**
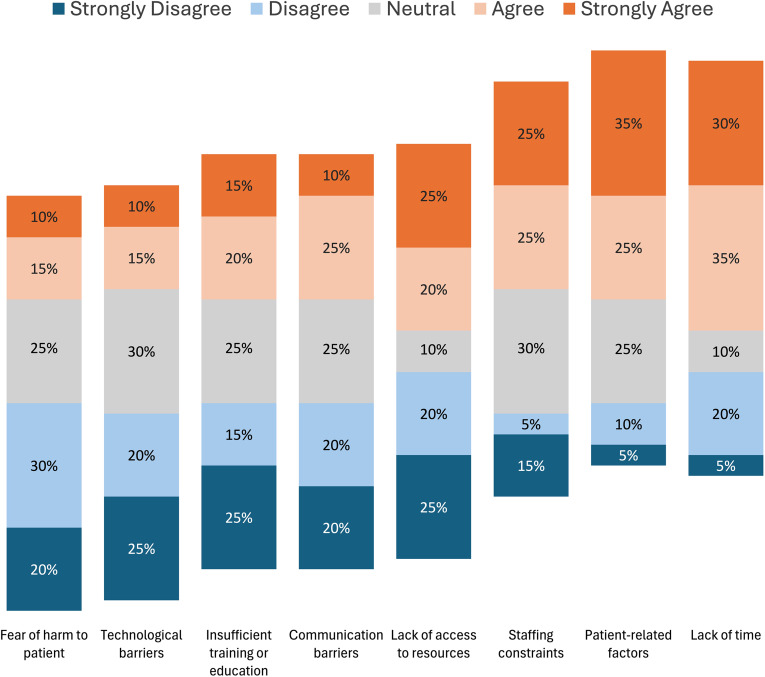
Survey results: ACE unit staff-reported barriers to penicillin allergy de-labeling (*n* = 20 respondents).

For the post-implementation survey, there were a total of 20 respondents consisting of clinical pharmacists (*n* = 5), pharmacy technicians (*n* = 3), physicians (*n* = 5), and nurses (*n* = 7). 65% of respondents either agreed or strongly agreed that lack of time was a barrier to implementation. Similarly, 60% and 50% of respondents either agreed or strongly agreed that patient-related factors such as clinical status/prognosis and staffing constraints, respectively, were other barriers (Figure 2). Fear of harm to patient, technological barriers, and insufficient training or education as barriers were less frequently reported (Figure 2).

**Table 2.** Outcomes

OUTCOMES	*n* (%)
**PRIMARY: **	
Eligible patients whose penicillin allergies were de-labeled	24/34 (71)
**SECONDARY:**	
Patients de-labeled by oral amoxicillin challenge:	14/24 (58)
No reaction	13/14 (93)
Immediate reaction	0
Delayed reaction within 4 weeks^a^	1/14 (7)
Patients de-labeled by information gathering alone^b^	10/24 (42)
Use of beta-lactam antibiotics within 4 weeks	10/24 (42)
Use of guideline-discordant broad-spectrum antibiotics within 4 weeks	0
Patients with updated allergy records based on new information	85/87 (98)

a Skin reaction with rib cage tightness and coughing after 7 days.

b Prior penicillin tolerance or reaction was intolerance (e.g. nausea, vomiting, etc.).

## Discussion

While penicillin allergy de-labeling has been done in various settings, data on elderly populations remain limited. Our pilot quality improvement study contributes to this gap in literature by specifically focusing on hospitalized elderly patients, who often have complex medical histories and a higher burden of antibiotic use. Our study population had a median age of 84 years, with a median Charlson Comorbidity Index of 6, indicating a high burden of chronic disease. In contrast, a number of previous de-labeling studies have either excluded patients aged 70 and older or included younger cohorts with lower comorbidity burdens.^14,19,22,24,25,27,36^ Furthermore, previous studies have not fully implemented comprehensive allergy assessments in a comorbid inpatient elderly population.^14,25,27,36^ A penicillin allergy de-labeling program implemented in Australia reported a 62% de-labeling rate for patients with a low-risk penicillin allergy, though this cohort had a lower median Charlson Comorbidity Index of 4 and a median age of 66.^22^ Similarly, the Veterans Affairs study population had a median age of 67 and a median Charlson Comorbidity Index score of 4.^24^ One study from the United States Drug Allergy Registry (USDAR) did apply allergy assessments to adults aged 65 years and up, but they report a mean age of 72, and the population in this registry was relatively healthy, without significant need for antibiotics.^27^ Similarly, the PALACE trial demonstrated the feasibility of direct oral amoxicillin challenge in low-risk adult patients who were relatively younger than our study population.^19^

Our initiative determined eligibility based on available clinical data and guidelines, and incorporated the PEN-FAST tool.^18,19,37,38^ While past research has characterized patients with a PEN-FAST score of ≤2 as low-risk in a large international randomized clinical trial, most penicillin challenge patients in the cohort had lower PEN-FAST scores of 0 or 1.^19^ As such, we opted for a more cautious de-labeling approach using a cut-off PEN-FAST score of 0–1 to balance safety for our elderly study population. In terms of outcomes, our de-labeling rate of 71% is comparable to previous studies, though our strict eligibility criteria may have contributed to a slightly lower de-labeling rate than some published reports.^25^ Ineligibilities to penicillin challenge included severe, recurrent, or multiple beta-lactam allergic reactions, unstable or poor clinical status, and concomitant use of immunosuppressive medications. Despite this, the safety of our approach was reinforced by the absence of immediate reactions and only one transient case of delayed hypersensitivity reaction.

It is important to note that piperacillin-tazobactam may express a distinct allergic phenotype compared to other penicillin or beta-lactam antibiotics.^39,40^ In some cases, individuals exhibit selective hypersensitivity reactions to piperacillin-tazobactam despite tolerating other penicillins. Given the limited evidence on penicillin allergy de-labeling in the elderly population, we excluded those with a documented piperacillin-tazobactam allergy from our study. Future studies may further explore piperacillin-tazobactam allergy phenotypes and cross-reactivity potential with other penicillins.

Our de-labeling efforts had positive implications for antimicrobial stewardship efforts, as 42% of de-labeled patients subsequently received beta-lactams, reducing reliance on guideline-discordant broad-spectrum antibiotics. These findings further support the integration of penicillin allergy assessments and de-labeling into routine care, especially in elderly patients who may have more frequent and prolonged hospitalization and infection risk.

The most frequently reported barriers by healthcare staff to implementation of this initiative were lack of time, patient complexity, and staffing constraints. Using our approach, standardized allergy assessment and oral challenge protocols may help with implementation efficiency. Further work is needed to address these concerns to ensure sustainability of penicillin allergy assessment and de-labeling programs.

Limitations of this study include its single-unit design, retrospective chart review, potential survey response bias. Additionally, we had limited access to outpatient antibiotic records for some patients after de-labeling. However, this likely would have been mitigated by the fact that all patients who were de-labeled with oral challenge were followed up by the clinical pharmacist after 4 weeks and were asked about any antibiotic use. Also, many de-labeled patients were still admitted in hospital 4 weeks following de-labeling so access to medication records would not have been a concern. Despite these limitations, our study was based on a comprehensive initiative utilizing information from patients, outpatient providers, health authority medical records, along with sufficient follow-up.

In summary, our quality improvement study highlights an effective and safe method to de-labeling penicillin allergies in a highly comorbid, elderly, inpatient population. By incorporating appropriate patient selection criteria (including PEN-FAST 0–1) and a structured approach, we provide a foundation for expanding penicillin allergy de-labeling protocols for older adults in similar hospital settings.

## Supporting information

10.1017/ash.2025.10279.sm001 Co et al. supplementary material 1 Co et al. supplementary material

10.1017/ash.2025.10279.sm002 Co et al. supplementary material 2 Co et al. supplementary material

10.1017/ash.2025.10279.sm003 Co et al. supplementary material 3 Co et al. supplementary material

## Supplementary material

The supplementary material for this article can be found at https://doi.org/10.1017/ash.2025.10279.

## References

[1] Luintel, A , Healy, J , Blank, M , Luintel, A , Dryden, S , Das, A , et al. The global prevalence of reported penicillin allergy: A systematic review and meta-analysis. J Infect 2025;90:106429. 39874990 10.1016/j.jinf.2025.106429

[2] Khan DA , Banerji A , Blumenthal KG , Phillips EJ , Solensky R , White AA , et al. Drug allergy: A 2022 practice parameter update. J Allergy Clin Immunol 2022;150:1333. 36122788 10.1016/j.jaci.2022.08.028

[3] Harandian F , Pham D , Ben-Shoshan M. Positive penicillin allergy testing results: a systematic review and meta-analysis of papers published from 2010 through 2015. Postgrad Med. 2016;128:557–62. 27240423 10.1080/00325481.2016.1191319

[4] Sacco KA , Bates A , Brigham TJ , Imam JS , Burton MC. Clinical outcomes following inpatient penicillin allergy testing: a systematic review and meta-analysis. Allergy. 2017;72:1288–96. 28370003 10.1111/all.13168

[5] Trubiano JA , Adkinson NF , Phillips EJ. Penicillin allergy is not necessarily forever. JAMA. 2017;318:82–3. 28672303 10.1001/jama.2017.6510 PMC5935455

[6] Blumenthal KG , Peter JG , Trubiano JA , Phillips EJ. Antibiotic allergy. Lancet. 2019;393:183–98. 30558872 10.1016/S0140-6736(18)32218-9 PMC6563335

[7] Blanca M , Torres MJ , García JJ , Romano A , Mayorga C , de Ramon E et al. Natural evolution of skin test sensitivity in patients allergic to beta-lactam antibiotics. J Allergy Clin Immunol. 1999;103:918–24. 10329829 10.1016/s0091-6749(99)70439-2

[8] Macy E , Contreras R. Health care use and serious infection prevalence associated with penicillin “allergy” in hospitalized patients: A cohort study. J Allergy Clin Immunol. 2014;133:790–6. 24188976 10.1016/j.jaci.2013.09.021

[9] Murphy J , Isaiah A , Dyalram D , Lubek JE. Surgical site infections in patients receiving osteomyocutaneous free flaps to the head and neck. Does choice of antibiotic prophylaxis matter? J Oral Maxillofac Surg. 2017;75:2223–9. 28282521 10.1016/j.joms.2017.02.006

[10] Sade K , Holtzer I , Levo Y , Kivity S. The economic burden of antibiotic treatment of penicillin-allergic patients in internal medicine wards of a general tertiary care hospital. Clin Exp Allerg. 2003;33:501–6. 10.1046/j.1365-2222.2003.01638.x 12680867

[11] Gray MP , Kellum JA , Kirisci L , Boyce RD , Kane-Gill SL. Long-term outcomes associated with β-lactam allergies. JAMA Netw Open. 2024;7:e2412313. 38758551 10.1001/jamanetworkopen.2024.12313 PMC11102016

[12] Lee CE , Zembower TR , Fotis MA , Postelnick MJ , Greenberger PA , Peterson LR et al. The incidence of antimicrobial allergies in hospitalized patients: implications regarding prescribing patterns and emerging bacterial resistance. Arch Int Med. 2000;160:2819–22. 11025792 10.1001/archinte.160.18.2819

[13] Blumenthal KG , Lu N , Zhang Y , Li Y , Walensky RP , Choi HK. Risk of meticillin resistant Staphylococcus aureus and Clostridium difficile in patients with a documented penicillin allergy: population based matched cohort study. BMJ. 2018;361:k2400. 29950489 10.1136/bmj.k2400 PMC6019853

[14] Gillespie C , Sitter K , McConeghy KW , Strymish J , Gupta K , Hartmann CW , et al. Facilitators and barriers to verifying penicillin allergies in a veteran nursing home population. J Allergy Clin Immunol Pract. 2023;11:2848–2854. 37352930 10.1016/j.jaip.2023.06.023

[15] McConeghy KW , Caffrey AR , Morrill HJ , Trivedi AN , LaPlante KL. Are non-allergic drug reactions commonly documented as medication “allergies”? A national cohort of Veterans’ admissions from 2000 to 2014. Pharmacoepidemiol Drug Saf. 2017;26:472–6. 27862587 10.1002/pds.4134

[16] Kline KA , Bowdish DME. Infection in an aging population. Curr Opin Microbiol. 2016;29:63–7. 26673958 10.1016/j.mib.2015.11.003

[17] Walker J , Wynne H. Review: the frequency and severity of adverse drug reactions in elderly people. Age Ageing. 1994;23:255–9. 8085514 10.1093/ageing/23.3.255

[18] Trubiano JA , Vogrin S , Chua KYL , Bourke J , Yun J , Douglas A , et al. Development and validation of a penicillin allergy clinical decision rule. JAMA Int Med. 2020;180:745–52. 10.1001/jamainternmed.2020.0403 PMC7076536 32176248

[19] Copaescu AM , Vogrin S , James F , Chua KYL , Rose MT , De Luca J , et al. Efficacy of a clinical decision rule to enable direct oral challenge in patients with low-risk penicillin allergy: the PALACE randomized clinical trial. JAMA Int Med. 2023;183:944–52. 10.1001/jamainternmed.2023.2986 PMC10352926 37459086

[20] Mill C , Primeau MN , Medoff E , Lejtenyi C , O’Keefe A , Netchiporouk E , et al. Assessing the diagnostic properties of a graded oral provocation challenge for the diagnosis of immediate and nonimmediate reactions to amoxicillin in children. JAMA Pediatr. 2016;170:e160033. 27043788 10.1001/jamapediatrics.2016.0033

[21] Tucker MH , Lomas CM , Ramchandar N , Waldram JD. Amoxicillin challenge without penicillin skin testing in evaluation of penicillin allergy in a cohort of Marine recruits. J Allergy Clin Immunol Pract. 2017;5:813–5. 28341170 10.1016/j.jaip.2017.01.023

[22] Chua KYL , Vogrin S , Bury S , Douglas A , Holmes NE , Tan N , et al. The penicillin allergy delabeling program: a multicenter whole-of-hospital health services intervention and comparative effectiveness study. Clin Infect Dis. 2021;73:487–96. 32756983 10.1093/cid/ciaa653 PMC8326579

[23] Kuruvilla M , Shih J , Patel K , Scanlon N. Direct oral amoxicillin challenge without preliminary skin testing in adult patients with allergy and at low risk with reported penicillin allergy. Allergy Asthma Proc. 2019;40:57–61. 30582497 10.2500/aap.2019.40.4184

[24] Arasaratnam RJ , Guastadisegni JM , Kouma MA , Maxwell D , Yang L , Storey DF. Rising to the challenge: An ID provider–led initiative to address penicillin allergy labels at a large Veterans Affairs medical center. Open Forum Infectious Diseases. 2024;11:ofae396. 39130085 10.1093/ofid/ofae396 PMC11310584

[25] Du Plessis T , Walls G , Jordan A , Holland DJ. Implementation of a pharmacist-led penicillin allergy de-labelling service in a public hospital. J Antimicrob Chemother. 2019;74:1438–46. 30753497 10.1093/jac/dky575

[26] Samarakoon U , Accarino J , Wurcel AG , Jaggers J , Judd A , Blumenthal KG. Penicillin allergy delabeling: opportunities for implementation and dissemination. Ann Allergy, Asthma Immunol. 2023;130:554–64. 36563744 10.1016/j.anai.2022.12.023 PMC11949300

[27] Accarino JJO , Ramsey A , Samarakoon U , Phillips E , Gonzalez-Estrada A , Otani IM , et al. Drug allergy in older adults: a study from the United States Drug Allergy Registry. Ann Allergy, Asthma Immunol. 2023;131:628–636. 37557950 10.1016/j.anai.2023.07.024

[28] Acute Care for the Elderly Unit (ACE) [Internet]. https://www.fraserhealth.ca/Service-Directory/Services/Hospital-Services/acute-care-for-the-elderly-unit. Accessed January 6, 2025.

[29] Burnaby Hospital [Internet]. https://www.fraserhealth.ca/Service-Directory/Locations/Burnaby/burnaby-hospital. Accessed February 3, 2025.

[30] Affiliated Teaching Sites|Anesthesiology, Pharmacology and Therapeutics [Internet]. https://apt.med.ubc.ca/hospital-sites/bc-teaching-sites/. Accessed February 3, 2025.

[31] Cane J , O’Connor D , Michie S. Validation of the theoretical domains framework for use in behaviour change and implementation research. Implement Sci. 2012;7:37. 22530986 10.1186/1748-5908-7-37 PMC3483008

[32] Lipworth W , Taylor N , Braithwaite J. Can the theoretical domains framework account for the implementation of clinical quality interventions? BMC Health Serv Res, 2013;13:530. 24359085 10.1186/1472-6963-13-530 PMC3901331

[33] Huijg JM , Gebhardt WA , Crone MR , Dusseldorp E , Presseau J. Discriminant content validity of a theoretical domains framework questionnaire for use in implementation research. Implement Sci. 2014;9:11. 24423394 10.1186/1748-5908-9-11 PMC3896680

[34] Phillips CJ , Marshall AP , Chaves NJ , Jankelowitz SK , Lin IB , Loy CT , et al. Experiences of using the Theoretical Domains Framework across diverse clinical environments: a qualitative study. J Multidiscip Healthc. 2015;8:139–46. 25834455 10.2147/JMDH.S78458 PMC4370908

[35] Atkins L , Francis J , Islam R , O’Connor D , Patey A Ivers N , et al. A guide to using the Theoretical Domains Framework of behaviour change to investigate implementation problems. Implement Sci. 2017;12:77. 28637486 10.1186/s13012-017-0605-9 PMC5480145

[36] Alagoz E , Saucke M , Balasubramanian P , Lata P , Liebenstein T , Kakumanu S. Barriers to penicillin allergy de-labeling in the inpatient and outpatient settings: a qualitative study. Allergy Asthma Clin Immunol. 2023;19:88. 37821953 10.1186/s13223-023-00842-y PMC10568923

[37] BC Provincial Antimicrobial Clinical Expert (PACE) Group. http://www.bccdc.ca/Documents/PACE%20Beta-lactam%20Allergy%20Delabeling%20Toolkit.pdf. Accessed December 21, 2024.

[38] American Academy of Allergy Asthma & Immunology. Penicillin-Allergy-Position-Statement_Approved-08-31-2023 [Internet], https://www.aaaai.org/Aaaai/media/Media-Library-PDFs/Allergist%20Resources/Statements%20and%20Practice%20Parameters/Penicillin-Allergy-Position-Statement_Approved-08-31-2023.pdf. Accessed 4 July 2024.

[39] Gallardo A , Moreno EM , Laffond E , Muñoz-Bellido FJ , Gracia-Bara MT , Macias EM , et al. Sensitization phenotypes in immediate reactions to piperacillin-tazobactam. J Allergy Clin Immunol: Pract. 2020;8:3175–7. 32320796 10.1016/j.jaip.2020.04.008

[40] Casimir-Brown RS , Kennard L , Kayode OS , Siew LQC , Makris M , Tsilochristou O , et al. Piperacillin-tazobactam hypersensitivity: a large, multicenter analysis. J Allergy Clin Immunol Pract. 2021;9:2001–9. 33444815 10.1016/j.jaip.2020.12.051

