# Supplementary material for: PEN-DEL: implementing penicillin allergy de-labeling in hospitalized older adults – a quality improvement initiative
Source: Antimicrob Steward Healthc Epidemiol. 2026 Feb 10;6(1):e44. doi: 10.1017/ash.2025.10279 (PMC12892141; doi:10.1017/ash.2025.10279)
Supplement: Co et al. supplementary material 1 — Co et al. supplementary material [file S2732494X25102799sup001.docx]

**Supplementary Material 1**

**
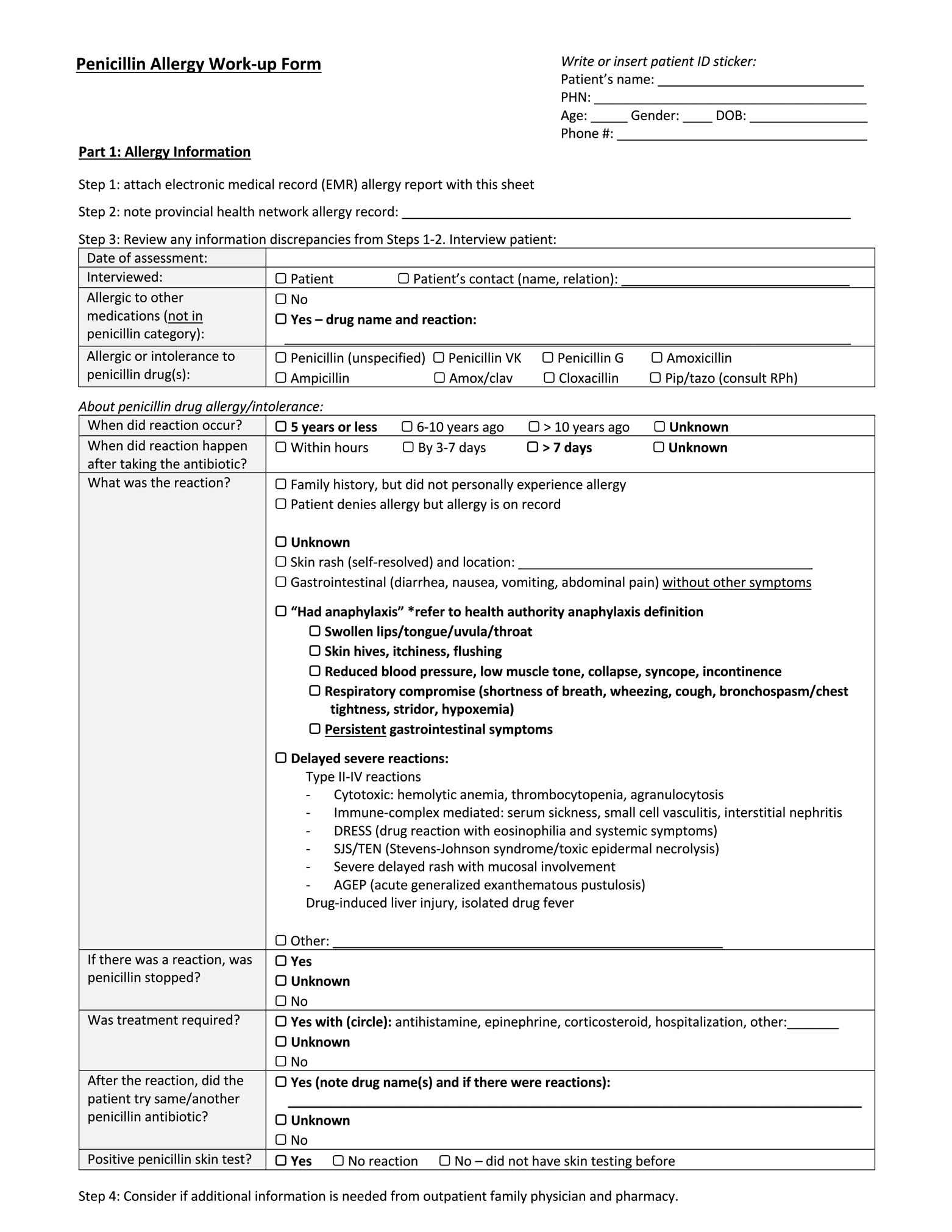
**

**PEN-FAST score^18^ calculation:** refer to the figure and figure notes in Trubiano JA, Vogrin S, Chua KYL, et al. Development and Validation of a Penicillin Allergy Clinical Decision Rule. JAMA Intern Med. 2020;180(5):745–752. doi:10.1001/jamainternmed.2020.0403

**Any contraindications to oral amoxicillin challenge:**

- NPO or unable to take oral medication
- Use of immunosuppressive medication (e.g., chronic prednisone equivalent of ≥15mg/day for ≥28 days)
- Significant cardiovascular disease (including active angina, NSTEMI, STEMI, CABG within last 6 months)
- Current oxygen supplementation ≥ 2 L/min (different from baseline)
- Clinically or hemodynamically unstable (SBP <100 mmHg)
- Asthma or COPD – active, uncontrolled
- Pregnant
- PEN-FAST >1
- Positive penicillin skin test
- Recurrent allergic reactions
- Type II-IV reaction, drug-induced liver/kidney injury, isolated drug fever
- Allergy to multiple beta-lactam antibiotics
- Anaphylaxis to any drug
- History of chronic spontaneous urticaria, mast cell disease
- Other reasons based on clinical judgement

*References for penicillin allergy assessment form: PEN-FAST tool^18^, PALACE RCT^19^, BC PACE guidelines^37^, and AAAAI update^38^.* Please refer to the manuscript for reference details.
