# Supplementary material for: PEN-DEL: implementing penicillin allergy de-labeling in hospitalized older adults – a quality improvement initiative
Source: Antimicrob Steward Healthc Epidemiol. 2026 Feb 10;6(1):e44. doi: 10.1017/ash.2025.10279 (PMC12892141; doi:10.1017/ash.2025.10279)
Supplement: Co et al. supplementary material 2 — Co et al. supplementary material [file S2732494X25102799sup002.docx]

Survey: Penicillin Allergy De-Labelling

1. **What is your current profession?** (select one)
   - Physician ☐ Nurse ☐ Pharmacist ☐ Pharmacy student/resident
   - Medical resident ☐ Pharmacy Technician ☐ Other (please specify):
2. **How many years have you been practicing?** (select one)

| - In training | - Less than 1 year | - 1-5 years | - 6-10 years |
| --- | --- | --- | --- |
| - 11-20 years | - More than 20 years |  |  |

| **Please rate how strongly you agree or disagree with each of the statements below:** | **1**  **Strongly disagree** | **2**  **Disagree** | **3**  **Neutral** | **4**  **Agree** | **5**  **Strongly agree** |
| --- | --- | --- | --- | --- | --- |
| 3. I am aware of the content and objectives  of penicillin allergy de-labelling | ◯ | ◯ | ◯ | ◯ | ◯ |
| 4. I am familiar with how to perform  penicillin allergy de-labelling tasks (e.g., allergy assessment, conducting oral  challenge, updating allergy records) | ◯ | ◯ | ◯ | ◯ | ◯ |
| 5. I have the skills to perform penicillin  allergy de-labelling tasks | ◯ | ◯ | ◯ | ◯ | ◯ |
| 6. Participating in penicillin allergy de-labelling is a part of my professional role | ◯ | ◯ | ◯ | ◯ | ◯ |
| 7. I am confident in my ability to conduct a penicillin allergy de-labelling task even  when there is little time | ◯ | ◯ | ◯ | ◯ | ◯ |
| 8. I believe that penicillin allergy de-labelling leads to improved patient outcomes (e.g., reduced antibiotic resistance, better  treatment options, fewer side effects) | ◯ | ◯ | ◯ | ◯ | ◯ |
| 9. I am motivated to engage in penicillin  allergy de-labelling as part of my standard workflow | ◯ | ◯ | ◯ | ◯ | ◯ |
| 10. I have enough time to incorporate penicillin allergy de-labelling into my  practice | ◯ | ◯ | ◯ | ◯ | ◯ |
| 11. I have the resources/supports I need to  perform penicillin allergy de-labelling | ◯ | ◯ | ◯ | ◯ | ◯ |

1. **Which healthcare professional do you think should take the lead on allergy de-labelling?** (select one)
   - Hospitalist ☐ Allergist ☐ Nurse ☐ Pharmacist ☐ GP ☐ Other (specify):
2. **Did you participate in penicillin allergy de-labelling in the ACE Unit?**
   - Yes ☐ No

*Please complete flip side 🡺*

1. **On average, how many minutes per patient did you spend (or expect to spend) on penicillin allergy de-labelling?** *Please provide your best estimate.* (i.e., time spent on screening, patient interviews, assessments, administering challenges, documentation, etc.)

minutes per patient

1. **To what extent do you believe the following impact your ability to participate in penicillin allergy de-labelling?** *Please rate each barrier on a scale of 1 to 5, where 1 = Not at all, and 5 = A great deal*

| **Barrier** | **1**  **(Not at all)** | **2** | **3**  **(Neutral)** | **4** | **5**  **(A great deal)** |
| --- | --- | --- | --- | --- | --- |
| Insufficient training or education | ◯ | ◯ | ◯ | ◯ | ◯ |
| Lack of time | ◯ | ◯ | ◯ | ◯ | ◯ |
| Staffing constraints | ◯ | ◯ | ◯ | ◯ | ◯ |
| Lack of access to necessary resources  (e.g., guidelines, PEN-FAST tool, PPO) | ◯ | ◯ | ◯ | ◯ | ◯ |
| Communication barriers within the  team | ◯ | ◯ | ◯ | ◯ | ◯ |
| Patient-related factors (e.g., patient  refusal, complex cases) | ◯ | ◯ | ◯ | ◯ | ◯ |
| Technological barriers (e.g., issues with  electronic records) | ◯ | ◯ | ◯ | ◯ | ◯ |
| Fear of harm to patient | ◯ | ◯ | ◯ | ◯ | ◯ |

1. **What other challenges do you anticipate in incorporating penicillin allergy de-labelling into your workflow?** *Please provide as much detail as possible.*
2. **How could the penicillin de-labelling process be improved in the future?**
