# Supplementary material for: PEN-DEL: implementing penicillin allergy de-labeling in hospitalized older adults – a quality improvement initiative
Source: Antimicrob Steward Healthc Epidemiol. 2026 Feb 10;6(1):e44. doi: 10.1017/ash.2025.10279 (PMC12892141; doi:10.1017/ash.2025.10279)
Supplement: Co et al. supplementary material 3 — Co et al. supplementary material [file S2732494X25102799sup003.docx]

**Supplementary Material 3: Additional Patient Baseline Characteristics**

| Characteristic | n = 87 |
| --- | --- |
| Received antibiotics during current admission, n (%)  Yes  No | 65 (75)  22 (25) |
| Carbapenems, n   - Ertapenem - Meropenem - Imipenem | 10  1  8  1 |
| Cephalosporins, n   - Cefazolin - Cephalexin - Cefuroxime - Cefixime - Ceftazidime - Ceftriaxone | 78  9  3  2  6  2  50 |
| Penicillins, n   - Penicillin VK - Penicillin G - Amoxicillin - Ampicillin - Amoxicillin-clavulanate - Cloxacillin - Piperacillin-tazobactam | 9  0  0  0  0  3  0  6 |
| Other, n | 41 |
| Concurrent cephalosporin allergy, n (%)  Ceftriaxone  Ceftazidime  Cefuroxime  Cephalexin | 7 (8)  1 (1)  1 (1)  2 (2)  2 (2) |
| Concurrent carbapenem allergy, n (%) | 0 |
| Concurrent non-beta-lactam antibiotic allergy, n (%) | 22 (25) |
| Concurrent non-antibiotic drug allergy, n (%) | 27 (31) |
| Admitted from long-term care/assisted living facility, n (%) | 25 (29) |
| Conditions during admission, n (%)  Infection-related  Non-infection-related | 51 (59)  36 (41) |
| Penicillin stopped if reaction experienced, n  Yes  No  Unknown  Patient denied penicillin allergy | 37 (42)  4 (5)  40 (46)  6 (7) |
| Same/another penicillin antibiotic tried after index penicillin reaction, n (%)  Yes   - Penicillin unspecified/VK   - Recurrent reaction - Amoxicillin   - Recurrent reaction - Amoxicillin-clavulanate - Ampicillin - Piperacillin-tazobactam   No  Unknown | 22 (25)  4 (5)  2  7 (8)  2  8 (9)  2 (2)  6 (7)  36 (41)  26 (30) |
